# Constrained Dynamic Matrix Control under International Electrotechnical Commission Standard 61499 and the Open Platform Communications Unified Architecture

**DOI:** 10.3390/s23156919

**Published:** 2023-08-03

**Authors:** Sergio Bustos-Pulluquitin, Gustavo Caiza, Mayra Llumitasig-Galarza, Maritza Castro-Mayorga, Clara Sánchez-Benítez, Marcelo V. Garcia

**Affiliations:** 1 Faculty of Natural Resources, Escuela Superior Politécnica de Chimborazo, Morona Santiago Campus, Macas 140101, Ecuador; patricio.bustos@espoch.edu.ec; 2 Carrera de Electrónica y Automatización, Universidad Politecnica Salesiana, Quito 170146, Ecuador; gcaiza@ups.edu.ec; 3 Department of Exact Sciences, Universidad de las Fuerzas Armadas, Latacunga Campus, Latacunga 050105, Ecuador; mcllumitasig@espe.edu.ec; 4 Faculty of Systems, Electronics and Industrial Engineering, Universidad Tecnica de Ambato, Ambato 180206, Ecuador; me.castro@uta.edu.ec (M.C.-M.); ca.sanchez@uta.edu.ec (C.S.-B.)

**Keywords:** Dynamic Matrix Control (DMC), IEC-61499, objective function, OPC-UA, optimization, predictive control

## Abstract

This paper focuses on the implementation of a constrained Dynamic Matrix Control (DMC) approach within the level processes of the FESTO™ MPS-PA Compact Workstation plant in the context of the Industrial Internet of Things (IIoT) paradigm. The goal is to develop an industrial control application with decentralized logic that optimizes the operation of the plant while adhering to specific constraints. The implementation is carried out using the IEC-61499 standard and the OPC-UA protocol, enabling seamless communication between devices and systems. The authors utilize the 4diac-IDE and 4diac-FORTE as the development and runtime environments, respectively, to enable the execution of the control application on low-cost devices. The Beagle Bone Black (BBB) card is used for data acquisition and actuator control. Three types of constraints are considered: control increment (Δu(k)), output (ym(k)), and control (u(k)) constraints, to prevent unnecessary stress on the actuator and avoid damage to the plant. The QP algorithm is employed to optimize the objective function and address these constraints effectively. By integrating advanced control strategies into industrial processes in the IIoT paradigm and implementing them on low-cost devices, this paper demonstrates the feasibility and effectiveness of improving system performance, resource utilization, and overall productivity while considering system limitations and constraints.

## 1. Introduction

As the global market becomes more unpredictable and competitive, manufacturing companies are faced with the challenge of enhancing the agility of their systems. To remain innovative and competitive, these companies must rapidly develop automated production processes that can adapt to changes efficiently. This necessitates the creation of large-scale industrial control systems and enterprise logistics systems that can incorporate change quickly. The interest in developing new technologies and architectures for the next generation of distributed systems in industrial automation has been growing steadily [1].

Traditionally, Programmable Logic Controllers (PLCs) have been widely used in industry due to their specific design for industrial applications. PLCs are tailored to control various systems through their inputs and outputs and they are programmed based on specific applications. However, there are challenges associated with the standardized programming languages for PLCs, such as IEC-61131-3. These challenges include complications arising from feedback connections within the application and difficulties in achieving communication between PLCs from different vendors [2].

In response to industry demands, there has been a shift from centralized systems like PLCs to more distributed paradigms in industrial automation. Large systems with centralized intelligence controlling every aspect are being transformed into distributed systems, where individual components possess intelligence and can communicate seamlessly with each other. This enables the system to act as a cohesive whole. The IEC-61499 standard defines a domain-specific modeling language that facilitates the development of distributed industrial control solutions. By extending the capabilities of IEC-61131-3, IEC-61499 improves software component encapsulation for enhanced reusability, provides a vendor-independent format and simplifies controller-to-controller communication. The distribution functionality and support for dynamic reconfiguration offered by IEC-61499 establish the necessary infrastructure for Industry 4.0 and Internet of Things (IoT) applications [3].

By leveraging the capabilities of the IEC-61499 standard and utilizing low-cost devices, this paper aims to address the control of processes through predictive control. This approach enables enhanced flexibility, reusability, and communication in distributed industrial control systems, aligning with the goals of Industry 4.0 and IoT. The paper expands on these concepts by highlighting the benefits and applications of IEC-61499 standard and the OPC-UA protocol, along with the growing adoption of advanced control systems like Model Predictive Control (MPC) in the industrial domain.

The utilization of the IEC-61499 standard in controlling processes offers significant advantages in terms of flexibility and reusability. By employing the IEC-61499 standard, the control logic can be encapsulated into software components that can be easily deployed and integrated into distributed industrial control systems. This modular approach allows for greater system design and reconfiguration flexibility, enabling the system to adapt to changing requirements and conditions. Additionally, the vendor-independent format provided by IEC-61499 facilitates interoperability and communication between controllers from different vendors, addressing the challenges associated with combining PLCs from various sources [4]. These capabilities make IEC-61499 an ideal choice for implementing decentralized control in the context of Industry 4.0 and IoT applications.

In the realm of advanced control techniques, MPCs stand out as a powerful method for optimizing process performance with its ability to handle multivariable control, constraints, and nonlinear processes [3,5]. By integrating MPCs into the control architecture based on the IEC-61499 standard, this paper aims to enhance the predictive control capabilities of the system. The utilization of MPCs enables the optimization of objective functions, such as minimizing process deviations or maximizing energy efficiency, while considering constraints imposed by the system’s dynamics, actuators, and other operational limitations. By combining the benefits of the IEC-61499 standard with MPCs, the proposed approach aims to enhance the efficiency and effectiveness of control strategies in industrial processes.

Furthermore, the adoption of the OPC-UA standard plays a crucial role in enabling seamless communication and interoperability in distributed industrial control systems. OPC-UA offers a platform-independent interface that facilitates data exchange and integration across various levels of the automation pyramid, from embedded systems to higher-level Manufacturing Execution Systems (MES) and Enterprise Resource Planning (ERP) systems [6]. By providing a standardized and robust communication protocol, OPC-UA ensures compatibility and facilitates the integration of diverse devices and software applications. The use of OPC-UA in the paper’s framework allows for efficient data acquisition and exchange between low-cost devices, such as sensors and actuators, and the control system. This integration enables real-time monitoring, analysis, and control of industrial processes, enhancing their overall performance and reliability.

In this approach, the integration of DMC into IEC-61499 Function Blocks (FBs), along with the utilization of OPC-UA for data exchange, offers several key advantages:

- Enhanced predictive capabilities: Incorporating MPCs within IEC-61499 FBs enables the control system to leverage the advanced predictive capabilities of MPCs. This allows for optimal control of multivariable processes, taking into account constraints and nonlinear dynamics;
- Modular and vendor-independent approach: The use of IEC-61499 FBs provides a modular and vendor-independent approach to control system design. This facilitates the flexible deployment and integration of DMC-based control strategies within distributed industrial systems, enhancing system flexibility and adaptability;
- Seamless interoperability: Leveraging OPC-UA for data communication ensures seamless interoperability between low-cost devices and the control system. OPC-UA enables real-time information exchange, facilitating efficient monitoring and control of industrial processes.

The aim of this article is the integration of MPCs into IEC-61499 FBs, along with the utilization of OPC-UA for data exchange, which represents a significant advancement in the field of industrial automation. This approach offers enhanced predictive capabilities, a modular and vendor-independent design and seamless interoperability with low-cost devices. By leveraging these technologies, manufacturing companies can achieve improved control performance, increased system flexibility, efficient monitoring and control of industrial processes.

This article is structured as follows: Section 2 presents different works related to the subject of study. Section 3 provides a comprehensive overview of key concepts and methodologies used in this research. Section 4 shows the case study for the research method and presents the implementation proposal of the MPC control algorithm using IEC-61499 and the monitoring using OPC-UA in low-cost devices. The results of the platform are shown in Section 5. The discussion of results is presented in Section 6. Finally, Section 7 details the conclusions and future work.

## 2. The State of the Art

Currently, the rapid development of technology such as Big Data, MES, smart sensors, and control systems have been reasons for Industry 4.0 to have a profound impact on industrial companies. Low-cost automation promotes cost-effective architectures and new development approaches to increase the flexibility and efficiency of production operations in an industrial plant. This has led to the adoption of standards and protocols such as IEC-61499 and OPC-UA to help companies integrate into Industry 4.0, which is why IEC-61499 and OPC-UA have been the subject of several research projects. In [7], we discuss: (a) how systems programmed in IEC-61131-3 are converted to IEC-61499-based systems; (b) how IEC-61499 has been integrated with distributed intelligent automation; and (c) how Integrated Development Environment (IDEs) for IEC-61499 have been implemented.

Traditionally, industrial control systems have relied on centralized architectures, with a central intelligence controlling all aspects of the system. This approach often leads to complex and rigid control systems that struggle to adapt to changing requirements and dynamic environments [8]. In contrast, the use of MPCs within the IEC-61499 standard offers a more distributed and flexible approach to control. By leveraging the concept of FBs, which encapsulate control logic and data, the IEC-61499 standard enables the development of modular and reusable control applications.

One of the key advantages of using MPCs within the IEC-61499 standard is the ability to handle multivariable control. MPCs are a model-based control technique that can simultaneously control multiple outputs, taking into account their interdependencies [9,10]. This capability allows for improved coordination and optimization of the control actions, leading to enhanced system performance and efficiency. In traditional approaches, coordinating control actions across multiple variables often requires extensive manual tuning and adjustments, leading to suboptimal performance and increased complexity.

Moreover, the integration of MPCs with the IEC-61499 standard facilitates seamless communication and interoperability between devices and systems through the use of the OPC-UA protocol. OPC-UA provides a standardized and secure framework for data exchange, ensuring compatibility between components from different vendors. This interoperability enables the integration of heterogeneous devices, sensors, and actuators into the control system, fostering the utilization of low-cost devices and enhancing the scalability of the overall system. In traditional control approaches, the lack of standardized communication protocols often hinders the integration of diverse devices and systems, limiting the flexibility and extensibility of the control system.

There are many control system applications at the academic level based on IEC-61499, as discussed in [11,12,13], where 4diac-FORTE has been implemented with low cost hardware, and FBs capable of encapsulating access to input, output, and control signals are provided. The application was implemented in a parts-handling station and a conveyor belt; likewise, in [14,15] implementations of load balancing applications using the IEC-61499 architecture are presented, the applications were performed in commercial programmable automation devices and embedded systems; likewise, in [16], models and concepts are presented to develop control systems for intelligent electrical networks based on holonic concepts and the open standards IEC-61850 and IEC-61499. There are also applications in the field of robotics where [17] proposes an implementation method of Cyber-Physical Production Systems (CPPSs) based on IEC-61499 for Physical Human-Robot Interaction (pHRI) and, finally, in [18] there is a distributed holonic control approach for a tire manufacturing system using FBs of the IEC-61499 standard.

Industry 4.0 introduces modern IT concepts in industrial contexts to create more flexible and innovative production processes. The fourth industrial revolution can only be achieved if sufficient effort is invested to introduce interoperability between industrial applications. Therefore OPC-UA plays a very important role within Industry 4.0; for such a reason, in [19] a solution towards OPC-UA and M2M interworking is proposed. Similarly, in [20], a CPPS architecture was implemented through a central OPC-UA server for Smart Manufacturing based on a 5G network.

The unification of IEC-61499 and OPC-UA will play a very important role in the 4.0 industry. In [21], an approach to accessing field data in automation systems using OPC-UA servers in CPPSs architectures is presented using IEC-61499 applications for industrial oil extraction processes. Similarly in [22] the design of a distributed control system embedded in a Raspberry Pi is presented, which delivers data to an OPC-UA server all under the IEC-61499 standard, and the application was implemented in a FESTO plant; also, in [23], the implementation of a classification system has been performed unifying the IEC-61499 and OPC-UA standards.

The MPC has been the subject of research and development for many years, there have been countless applications in both industry and education; for example, [24] has dealt with the teaching, practice, and application of a level MPC; similarly, [25] presents the latest developments of MPCs for power converters and drives, and also describes the current status of this strategy and analyzes new trends. Another field of application is given in [26], where a general description of MPC stability results is provided; and finally, in [27], a general description of the main developments and challenges in the MPCs area is given.

In summary, the adoption of DMC within the IEC-61499 standard brings significant advantages compared to traditional control approaches. It enables multivariable control, dynamic reconfiguration, and seamless interoperability through standardized communication protocols. These features empower industrial control systems with enhanced performance, flexibility, and adaptability, making them well-suited for the requirements of modern manufacturing processes within the IIoT paradigm. The combination of DMC, IEC 61499, and OPC-UA represents a powerful framework for developing advanced control applications that can optimize system performance, energy consumption, and overall productivity, while maintaining system integrity and ensuring compliance with operational constraints.

## 3. Technical Background

The Technical Background section provides a comprehensive overview of key concepts and methodologies in the field of automation and control of processes. This section encompasses several subtopics that are integral to understanding the intricacies of this domain. Firstly, the IEC’s standard IEC-61499 is explored, which lays the foundation for distributed control systems and event-driven architectures. The next subtopic delves into OPC-UA, a robust communication protocol that enables seamless interoperability between various automation devices and software applications. Moving forward, MPC is discussed, highlighting its significance in optimizing system performance by predicting future behavior and utilizing feedback loops. Additionally, the section covers MC, a powerful algorithm widely employed for real-time optimization of complex processes. Lastly, the General Quadratic Programming subtopic elucidates the mathematical optimization technique used to solve various control problems by minimizing or maximizing a quadratic objective function. Together, these subtopics provide a solid technical foundation for understanding the subsequent research findings.

### 3.1. IEC-61499

The IEC-61499 architecture presents a component-based solution for distributed industrial automation systems with the objective of achieving portability, reusability, interoperability, and reconfiguration of distributed applications. Within this framework, IEC-61499 establishes a generic model that encompasses processes and communication networks, providing an environment for embedded devices, resources, and applications. Applications are constructed by assembling networks of Function Blocks (FBs) [28]. IEC-61499 introduces a modeling language similar to traditional FBs but tailored to distributed systems, thereby enabling the modeling of entire systems, even if they consist of smaller constituents like individual PLCs and embedded systems. The application is then built by interconnecting these individual FBs. Furthermore, the standard defines a model to represent the devices in a system and their interconnections. In cases where the application spans multiple devices, all FBs in the application can be mapped to their respective devices [2].

**Figure 1:**
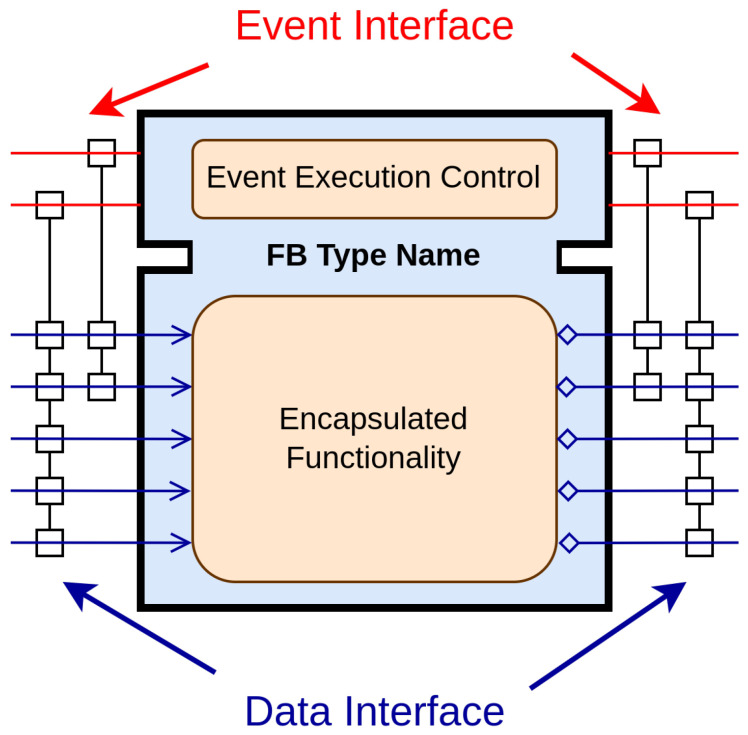
IEC-61499 function block.

Figure 1 depicts an FB in the context of IEC-61499. The FB encapsulates the desired functionality, but unlike the FBs in IEC-61131-3, it distinguishes between events and data, where events trigger the functionalities of the FBs. Each event input is connected to several data inputs, and each event output is connected to several data outputs. The behavior of the FB is governed by the Event Execution Control (ECC), which functions as a state machine that responds to input events.

**Figure 2:**
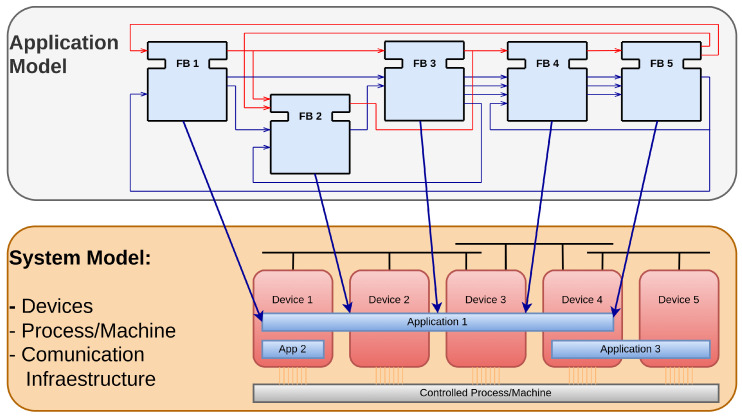
Distributed application model in IEC-61499.

In Figure 2, a distributed application model is represented, showcasing the flexibility of IEC-61499. Unlike traditional approaches, this model allows the application to be split and deployed across multiple devices such as PLCs, BBB, and Raspberry Pi (RPI). Moreover, this distributed architecture enables the deployment of multiple applications, further enhancing the scalability and adaptability of the system.

### 3.2. OPC-UA

OPC-UA represents a significant advancement from the original OPC standard, which was limited to Microsoft Windows devices. OPC-UA, on the other hand, was specifically designed to harness the full potential of modern technologies in creating smart factories, encompassing mobile devices, large databases, machine learning, machine vision, artificial intelligence, deep learning, and predictive maintenance, among others [29]. With the multitude of machines, devices and systems within modern manufacturing and logistics operations, OPC-UA serves as the vital link to connect these isolated islands. Its key requirements include reliable communication between distributed systems, ensuring robustness, fault tolerance, platform independence, scalability, high performance, compatibility with the internet and firewalls, security, access control, and interoperability. Another crucial aspect is the modeling of data, which involves establishing a common model for all OPC data, utilizing an object-oriented approach, employing an extensible type system, incorporating metainformation, supporting complex data and methods, facilitating scalability from simple to complex models, establishing an abstract base model, and serving as the foundation for other standard data models.

**Figure 3:**
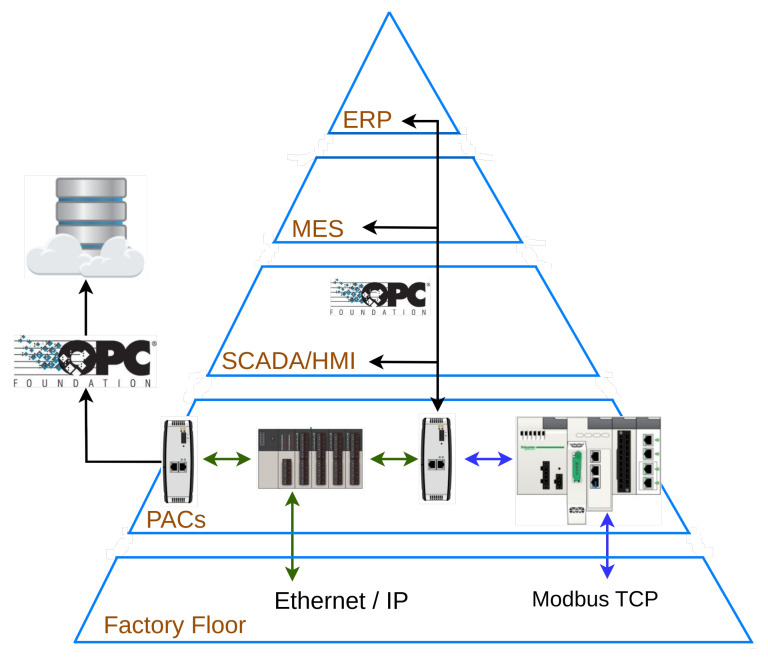
Automation pyramid and the use of OPC-UA to integrate all the floors.

In conjunction with OPC-UA, IEC-61499 provides a distributed application model that enhances the flexibility and scalability of automation and control systems. The distributed application model allows the deployment of applications across multiple devices, such as Programmable Logic Controllers (PLCs), BeagleBone Black (BBB), and Raspberry Pi (RPI) (see Figure 3). This distributed architecture accommodates the increasing complexity and interconnectedness of modern industrial systems, offering a versatile approach to managing and controlling various processes. By combining the power of OPC-UA for seamless communication and interoperability and the distributed application model of IEC-61499, manufacturers can effectively integrate diverse devices, systems, and technologies, optimizing their operations and paving the way for the realization of smart factories and efficient logistics operations [30].

One significant advantage of OPC-UA is its ability to be implemented in low-cost devices, revolutionizing the accessibility of advanced automation and control capabilities [31]. Traditionally, sophisticated industrial protocols were limited to high-end hardware, creating barriers for small-scale or budget-constrained applications. However, OPC-UA breaks this barrier by enabling the integration of low-cost devices into the industrial ecosystem. This opens up new possibilities for cost-effective automation solutions, allowing small businesses and organizations with limited resources to leverage the benefits of OPC-UA for efficient data exchange, interoperability, and real-time control. By extending OPC-UA to low-cost devices, a wider range of industries and applications can embrace the advantages of modern automation technologies, contributing to increased productivity, streamlined operations, and improved overall performance.

### 3.3. Model Predictive Control

The integration of MPCs into the FBs of the IEC-61499 standard introduces powerful control capabilities within the distributed application model. MPC, a robust control strategy, enables the prediction of system behavior based on dynamic models and optimization algorithms [32]. By incorporating MPCs into FBs, the IEC-61499 standard empowers control engineers to develop advanced control solutions that can handle complex process dynamics and optimize system performance. The Function Blocks act as modular components that encapsulate the MPC algorithms, enabling their seamless integration into the distributed application architecture [33]. This integration facilitates the deployment of MPC-based control strategies across multiple devices, providing a distributed control system capable of real-time optimization and adaptive control. With the incorporation of MPC into Function Blocks, the IEC-61499 standard enhances the capabilities of automation and control systems, enabling more efficient and intelligent management of processes across diverse industries.

Model Predictive Control (MPC) encompasses a wide range of control techniques that explicitly utilize a process model to derive the control signal by minimizing an objective function [34]. The fundamental ideas shared across all predictive control approaches include:

- Utilizing a process model to predict the future output of the system over a certain time horizon;
- Calculating a control sequence that optimizes an objective function based on the predicted system behavior;
- Implementing a receding strategy, whereby the time horizon is continually shifted forward at each time step, and only the first control signal from the calculated sequence is applied.

**Figure 4:**
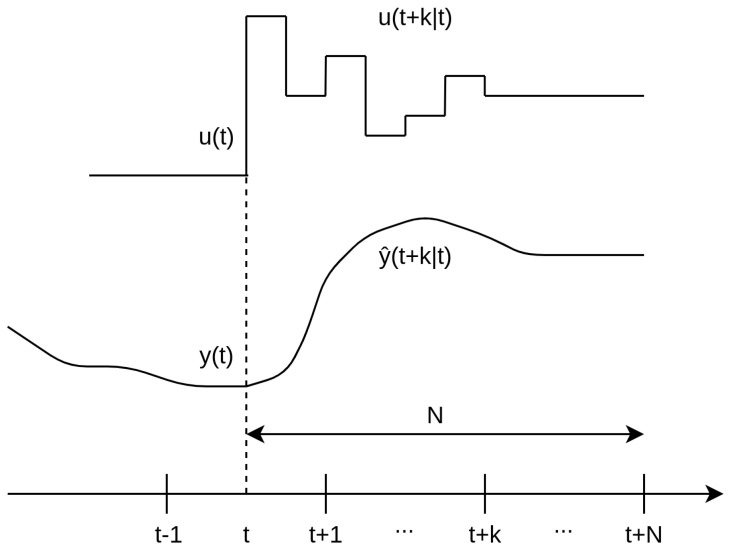
MPC strategy.

All MPC algorithms share essential components, such as the prediction model, objective function, and control law derivation. The methodology of all controllers belonging to the MPC family is characterized by the strategic process depicted in Figure 4.

1. At each time instant *t*, the process model is used to predict the future outputs for a specified prediction horizon *N*. These predicted outputs, denoted as $y{\left(t+k|t\right)}^{1}$ for k=1⋯N, depend on the known values up to time *t* (past inputs and outputs) and on the future control signals u(t+k|t), where k=0⋯N−1. These control signals are both sent to and calculated for the system [34]. Here, the notation indicates the value of the variable at time instant t+k, calculated at time *t*;
2. The set of future control signals is determined by optimizing a specific criterion, aiming to minimize the discrepancy between the process behavior and a reference trajectory w(t+k). Typically, this criterion takes the form of a quadratic function, utilizing the errors between the predicted output signal and the predicted reference trajectory [34]. In most cases, the objective function also considers the control effort;
3. The control signal u(t|t) is applied to the process while the subsequent calculated control signals are not immediately applied. Instead, at the next sampling instant, y(t+1) is known, and the prediction process is repeated with this new value, updating all control signal sequences. This concept is known as the receding horizon approach [34].

**Figure 5:**
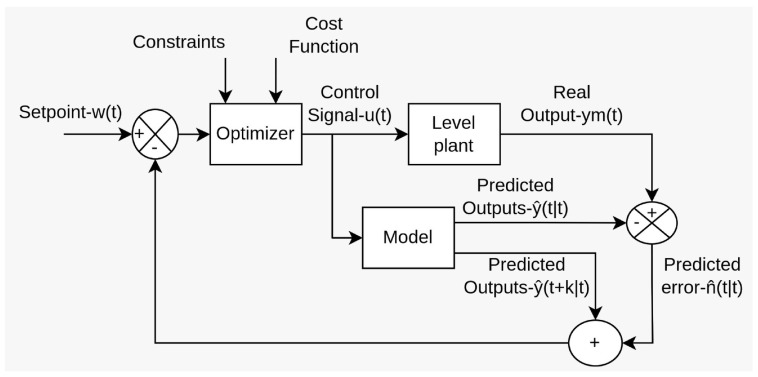
Basic structure of MPC.

In order to implement this strategy, the basic structure shown in Figure 5 is used.

#### Dynamic Matrix Control

As step response model employed is $y\left(t\right)=\sum_{i=1}^{\infty }g_{i}\Delta u\left(t-i\right)$ where: $g_{i}$ are the coefficients to step response and Δu are the control variations. The predicted values along the horizon will be [34]:

$$\hat{y}\left(t+k|t\right)=\sum_{i=1}^{\infty }g_{i}\Delta u\left(t+k-i\right)+\hat{n}\left(t+k|t\right)\;=\sum_{i=1}^{k}g_{i}\Delta u\left(t+k-i\right)+\sum_{i=k+1}^{\infty }g_{i}\Delta u\left(t+k-i\right)+\hat{n}\left(t+k|t\right)$$

Disturbances are considered to be constant, i.e., $\hat{n}\left(t+k|t\right)=\hat{n}\left(t|t\right)=y_{m}\left(t\right)-\hat{y}\left(t|t\right)$. Then it can be written that:

$$\hat{y}\left(t+k|t\right)=\sum_{i=1}^{k}g_{i}\Delta u\left(t+k-i\right)+\sum_{i=k+1}^{\infty }g_{i}\Delta u\left(t+k-i\right)\;+y_{m}\left(t\right)-\sum_{i=1}^{\infty }g_{i}\Delta u\left(t-i\right)=\sum_{i=1}^{k}g_{i}\Delta u\left(t+k-i\right)+f\left(t+k\right)$$

where f(t+k) is the free response of the system, that is, the part of the response that does not depend on the future control actions and is given by [34]:

$$f\left(t+k\right)=y_{m}\left(t\right)+\sum_{i=1}^{\infty }\left(g_{k+i}-g_{i}\right)\Delta u\left(t-i\right)$$

The coefficients $g_{i}$ of the step response tend to a constant value after *N* sampling periods, so it can be considered that $g_{k+i}-g_{i}\approx 0$ when i>N and therefore the free response can be computed as [34]:

$$f\left(t+k\right)=y_{m}\left(t\right)+\sum_{i=1}^{N}\left(g_{k+i}-g_{i}\right)\Delta u\left(t-i\right)$$

Now, the predictions can be computed along the prediction horizon (k=1,⋯,P), considering *N* control actions.

$$\hat{y}\left(t+1|t\right)=g_{1}\Delta u\left(t\right)+f\left(t+1\right)\;\hat{y}\left(t+2|t\right)=g_{1}\Delta u\left(t+1\right)+g_{2}\Delta u\left(t\right)+f\left(t+2\right)\;\hat{y}\left(t+3|t\right)=g_{1}\Delta u\left(t+2\right)+g_{2}\Delta u\left(t+1\right)+g_{3}\Delta u\left(t\right)+f\left(t+3\right)\vdots \;\vdots \;\hat{y}\left(t+P|t\right)=\sum_{i=P-N+1}^{P}g_{i}\Delta u\left(t+P-i\right)+f\left(t+P\right)$$

it can be written that [34]:

(1) $$\hat{y}=G\Delta u+f\;\left[\begin{array}{c} \hat{y}\left(t+1|t\right)\\ \hat{y}\left(t+2|t\right)\\ \vdots \\ \hat{y}\left(t+P|t\right) \end{array}\right]=\left[\begin{array}{cccc} g_{1} & 0 & \cdots & 0\\ g_{2} & g_{1} & \cdots & 0\\ \vdots & \vdots & \ddots & \vdots \\ g_{N} & g_{N-1} & \cdots & g_{1}\\ \vdots & \vdots & \ddots & \vdots \\ g_{P} & g_{P-1} & \cdots & g_{P-N+1} \end{array}\right]\left[\begin{array}{c} \Delta u(t)\\ \Delta u(t+1)\\ \vdots \\ \Delta u(t+N-1) \end{array}\right]+\left[\begin{array}{c} f(t+1)\\ f(t+2)\\ \vdots \\ f(t+P) \end{array}\right]$$

The objective of a DMC controller is to drive the output as close to the setpoint as possible in a least-squares sense with the possibility of the inclusion of a penalty term on the input moves. Therefore, the manipulated variables are selected to minimize a quadratic objective that consider the minimization of future errors and the control effort [34].

(2) $$J=\sum_{j=1}^{P}\delta \left(j\right){\left[\hat{y}\left(t+j|t\right)-w\left(t+j\right)\right]}^{2}+\sum_{j=1}^{N}\lambda \left(j\right){\left[\Delta u\left(t+j-i\right)\right]}^{2}$$

where the weighting sequences δ(j) and λ(j) are usually chosen constant and the reference trajectory w(t+j) can be generated by a simple recursion which starts at the current output and tends exponentially to the setpoint. Equation (2) written in matrix form will be

$$J={\left(\hat{y}-w\right)}^{T}I_{\delta }\left(\hat{y}-w\right)+\Delta u^{T}I_{\lambda }\Delta u$$

Replacing values by Equation (1) we have:

(3) $$J={\left(Gu+f-w\right)}^{T}I_{\delta }\left(Gu+f-w\right)+\Delta u^{T}I_{\lambda }\Delta u$$

If there are no constraints, the solution to the minimization of the cost function is obtained by deriving Equation (3) and equating to zero $\frac{\partial J}{\partial \Delta u}=0$:

(4) $$\Delta u={\left(G^{T}I_{\delta }G+I_{\lambda }\right)}^{-1}G^{T}I_{\delta }\left(w-f\right)=K\left(w-f\right)$$

If there are constraints the solution to the minimization of the cost function swill be by implementing a quadratic optimization algorithm. From Equation (3) we have:

(5) $$J=\Delta u^{T}\left(G^{T}I_{\delta }G+I_{\lambda }\right)\Delta u+2{\left(f-w\right)}^{T}I_{\delta }G\Delta u+{\left(f-w\right)}^{T}I_{\delta }\left(f-w\right)$$

and comparing to the matrix quadratic equation $J=\frac{1}{2}\Delta u^{T}H\Delta u+b^{T}\Delta u+f_{0}$ we have:

(6) $$H=2G^{T}I_{\delta }G+I_{\lambda }$$

(7) $$b^{T}=2{\left(f-w\right)}^{T}I_{\delta }G\;f_{0}={\left(f-w\right)}^{T}I_{\delta }\left(f-w\right)$$

The control problem has been formulated in Equation (4) considering all signals to possess an unlimited range, but in practice all processes are subject to constraints. Actuators have a limited range of action and a limited slew rate, for constructive and/or safety reasons, as well as sensor range, and cause bounds in process variables, as in the case of levels in tanks, flows in pipes, and pressures in deposits [34].

- The constraint in the increase in control Δu(k) is defined as $\Delta u_{min}\leq \Delta u\leq \Delta u_{max}$.

$$\left[\begin{array}{c} I\\ \cdots \\ -I \end{array}\right]\Delta u\leq \left[\begin{array}{c} 1\Delta u_{max}\\ \cdots \\ -1\Delta u_{min} \end{array}\right]$$

where *I* is an identity matrix of $N_{u}\times N_{u}$ and 1 is a column vector filled with 1s and dimensions $N_{u}\times 1$.

$$\left[\begin{array}{cccc} 1 & 0 & \cdots & 0\\ 0 & 1 & \cdots & 0\\ \vdots & \vdots & \ddots & \vdots \\ 0 & 0 & \cdots & 1\\ -1 & 0 & \cdots & 0\\ 0 & -1 & \cdots & 0\\ \vdots & \vdots & \ddots & \vdots \\ 0 & 0 & \cdots & -1 \end{array}\right]\left[\begin{array}{c} \Delta u(k)\\ \Delta u(k+1)\\ \vdots \\ \Delta u(k+N_{u}-1)\\ \Delta u(k)\\ \Delta u(k+1)\\ \vdots \\ \Delta u(k+N_{u}-1) \end{array}\right]\leq \left[\begin{array}{c} \Delta u_{max}\\ \Delta u_{max}\\ \vdots \\ \Delta u_{max}\\ -\Delta u_{min}\\ -\Delta u_{min}\\ \vdots \\ -\Delta u_{min} \end{array}\right]$$

- The constraint on the control signal u(k) is defined as $u_{min}\leq u\left(k\right)\leq u_{max}$; furthermore, Δu(k)=u(k)−u(k−1) replacing Δu(k) we have $u_{min}u\left(k\right)+u\left(k-1\right)u_{max}$.

$$\left[\begin{array}{c} T\\ \cdots \\ -T \end{array}\right]\Delta u\leq \left[\begin{array}{c} 1u_{max}-1u\left(k-1\right)\\ \cdots \\ -1u_{min}+1u\left(k-1\right) \end{array}\right]$$

where T represents a lower triangular matrix of dimension $N_{u}\times N_{u}$ and 1 is a 1s filled column vector of dimension $N_{u}\times 1$.

$$\left[\begin{array}{cccc} 1 & 0 & \cdots & 0\\ 1 & 1 & \cdots & 0\\ \vdots & \vdots & \ddots & \vdots \\ 1 & 1 & \cdots & 1\\ -1 & 0 & \cdots & 0\\ -1 & -1 & \cdots & 0\\ \vdots & \vdots & \ddots & \vdots \\ -1 & -1 & \cdots & -1 \end{array}\right]\left[\begin{array}{c} \Delta u(k)\\ \Delta u(k+1)\\ \vdots \\ \Delta u(k+N_{u}-1)\\ \Delta u(k)\\ \Delta u(k+1)\\ \vdots \\ \Delta u(k+N_{u}-1) \end{array}\right]\leq \left[\begin{array}{c} u_{max}-u\left(k-1\right)\\ u_{max}-u\left(k-1\right)\\ \vdots \\ u_{max}-u\left(k-1\right)\\ -u_{min}+u\left(k-1\right)\\ -u_{min}+u\left(k-1\right)\\ \vdots \\ -u_{min}+u\left(k-1\right) \end{array}\right]$$

- The plant output constraint is defined as $y_{min}\leq \hat{y}\leq y_{max}$; replacing the value of Equation (1), we have $y_{min}\leq G\Delta u+f\leq y_{max}$.

$$\left[\begin{array}{c} G\\ \cdots \\ -G \end{array}\right]\Delta u\leq \left[\begin{array}{c} 1y_{max}-f\\ \cdots \\ -1y_{min}+f \end{array}\right]$$

where *G* has dimensions $P\times N_{u}$ and 1 is a column vector filled with 1s and dimensions P×1.

$$\left[\begin{array}{cccc} g_{1} & 0 & \cdots & 0\\ g_{2} & g_{1} & \cdots & 0\\ \vdots & \vdots & \ddots & \vdots \\ g_{N} & g_{N-1} & \cdots & g_{1}\\ \vdots & \vdots & \ddots & \vdots \\ g_{P} & g_{P-1} & \cdots & g_{P-N+1}\\ -g_{1} & 0 & \cdots & 0\\ -g_{2} & -g_{1} & \cdots & 0\\ \vdots & \vdots & \ddots & \vdots \\ -g_{N} & -g_{N-1} & \cdots & -g_{1}\\ \vdots & \vdots & \ddots & \vdots \\ -g_{P} & -g_{P-1} & \cdots & -g_{P-N+1} \end{array}\right]\left[\begin{array}{c} \Delta u(k)\\ \Delta u(k+1)\\ \vdots \\ \Delta u(k+N_{u}-1)\\ \Delta u(k)\\ \Delta u(k+1)\\ \vdots \\ \Delta u(k+N_{u}-1) \end{array}\right]\leq \left[\begin{array}{c} y_{max}-f\left(k\right)\\ y_{max}-f\left(k\right)\\ \vdots \\ y_{max}-f\left(k\right)\\ -y_{min}+f\left(k\right)\\ -y_{min}+f\left(k\right)\\ \vdots \\ -y_{min}+f\left(k\right) \end{array}\right]$$

### 3.4. General Quadratic Programming (QP)

Consider the model problem

(8) $$\left.\begin{array}{cc} \mathrm{minimize}:\;c^{T}x+\frac{1}{2}x^{T}Cx & \\ \mathrm{subject}\;\mathrm{to}:\;a_{i}x\leq b_{i},\;\;i=1,\cdots ,m \end{array}\right\}$$

Assume that **C** is positive semidefinite and that $a_{1},\cdots ,a_{m}$ are linearly independent. Let $f\left(x\right)=c^{T}x+\frac{1}{2}x^{T}Cx$. We formulate an algorithm for the solution of Equation (8), which constructs a sequence of points $x_{0}$, $x_{1}$, $x_{2}$, ... according to $x_{j+1}=x_{j}-$σ${}_{j}s_{j},j=0,1,\cdots$ and, after finitely many steps, either determine an optimal solution for Equation (8) or determine that Equation (8) is unbounded from below. we will call $s_{j}$ and σ${}_{j}$ the search direction and step size, respectively, at iteration *j*. For any x∈R [35], let

$$I\left(x\right)=\left\{i|a_{i}^{T}x=b_{i},1\leq i\leq m\right\}$$

so that I(x) denotes the (unordered) set of indices of constraints active at x. By definition, each of 1,⋯,m is in I(x). At iteration *j*, let $K_{j}$ denote an ordered subset of $I(x_{j})$ such that $a_{i}$, all $i\in K_{j}$, are linearly independent. We refer to $K_{j}$ as the active set at iteration *j*.

In order to initiate the algorithm, we require a feasible point $x_{0}$ and $K_{0}\subseteq I\left(x_{0}\right)$ satisfying Assumption 1.

(i) $a_{i}$ all $i\in K_{0}$ are linearly independent;
(ii) $s^{\prime }Cs>0$ for all s≠0 with $A_{0}s=0$.

As a consequence of Assumption 1, we will show that $H_{0}$ is nonsingular. Note that Assumption 1(ii) would be satisfied if $x_{0}$ were a nondegenerate extreme point. Furthermore, Assumption 1(ii) would be satisfied if C is positive definite. A detailed statement of the algorithm follows [35].

#### QP Algorithm

Model problem:

$$\mathrm{minimize}:\;c^{T}x+\frac{1}{2}x^{T}Cx\;\;\;\;\;\;\;$$

$$\mathrm{subject}\;\mathrm{to}:\;a_{i}^{T}x\leq b_{i},\;\;i=1,\cdots ,m$$

Initialization:

Start with any feasible point $x_{0}$ and active set $K_{0}\subseteq I\left(x_{0}\right)$ such that Assumption 1 is satisfied. Compute $f\left(x_{0}\right)=c^{T}x_{0}+\frac{1}{2}x_{0}^{T}Cx_{0}$, $g_{0}=c+Cx_{0}$ and set j=0.

**Step 1**: Computation of Search Direction $s_{j}$.

Let

$$H_{j}=\left[\begin{array}{cc} C & A_{j}^{T}\\ A_{j} & 0 \end{array}\right].$$

If $H_{j}$ is nonsingular, go to Step 1.1. Otherwise, go to Step 1.2.

**Step 1.1**:

Compute the Newton direction $s_{j}$ and multipliers $v_{j}$ from the solution of the linear equations

$$H_{j}\left[\begin{array}{c} s_{j}\\ v_{j} \end{array}\right]=\left[\begin{array}{c} g_{j}\\ 0 \end{array}\right].$$

Set $\gamma_{j}=0$ and go to Step 2.

**Step 1.2**:

Compute $s_{j}$ such that $A_{j}s_{j}=0$, $Cs_{j}=0$, and $a_{k}^{T}s_{j}=1$. Set $\gamma_{j}=1$ and go to Step 2.

**Step 2**: Computation of Step Size σ${}_{j}$.

Set ${\tilde{\sigma }}_{j}=1$ if $\gamma_{j}=0$ and ${\tilde{\sigma }}_{j}=+\infty$ if $\gamma_{j}=1$. If $a_{j}^{T}s_{j}\geq 0$ for i=1,⋯,m, set ${\tilde{\sigma }}_{j}=+\infty$. Otherwise, compute the smallest index l and ${\tilde{\sigma }}_{j}$ such that

$${\tilde{\sigma }}_{j}=\frac{a_{l}^{T}x_{j}-b_{l}}{a_{l}^{T}s_{j}}=\min\left\{\frac{a_{i}^{T}x_{j}-b_{i}}{a_{i}^{T}s_{j}}|\;\mathrm{all}\;i\notin K_{j}\;\mathrm{with}\;a_{i}^{T}s_{j}<0\right\}.$$

If ${\tilde{\sigma }}_{j}={\hat{\sigma }}_{j}=+\infty$, print the message “the objective function is unbounded from below” and stop. Otherwise, set $\sigma_{j}=\min\left\{{\tilde{\sigma }}_{j},{\hat{\sigma }}_{j}\right\}$ and go to Step 3.

**Step 3**: Update.

Set $x_{j+1}=x_{j}-\sigma_{j}s_{j}$, $g_{j+1}=c+Cx_{j+1}$, and $f\left(x_{j+1}\right)=c^{T}x_{j+1}+\frac{1}{2}x_{j+1}^{T}Cx_{j+1}$. If $\sigma_{j}={\hat{\sigma }}_{j}$, go to Step 3.1. Otherwise, go to Step 3.2.

**Step 3.1**:

Set $K_{j+1}=K_{j}+l$, form $A_{j+1}$ and $H_{j+1}$, replace *j* with j+1 and go to Step 1.1.

**Step 3.2**:

Compute the multiplier vector $u_{j+1}$ from $-v_{j}$ and $K_{j}$ and compute k such that

$${\left(u_{j+1}\right)}_{k}=\min\left\{{\left(u_{j+1}\right)}_{i}|i=1,\cdots ,m\right\}.$$

If ${\left(u_{j+1}\right)}_{k}\geq 0$, then stop with optimal solution $x_{j+1}$. Otherwise, set $K_{j+1}=K_{j}-k$, form $A_{j+1}$ and $H_{j+1}$, replace *j* with j+1 and go to Step 1 [35].

## 4. Case Study

**Figure 6:**
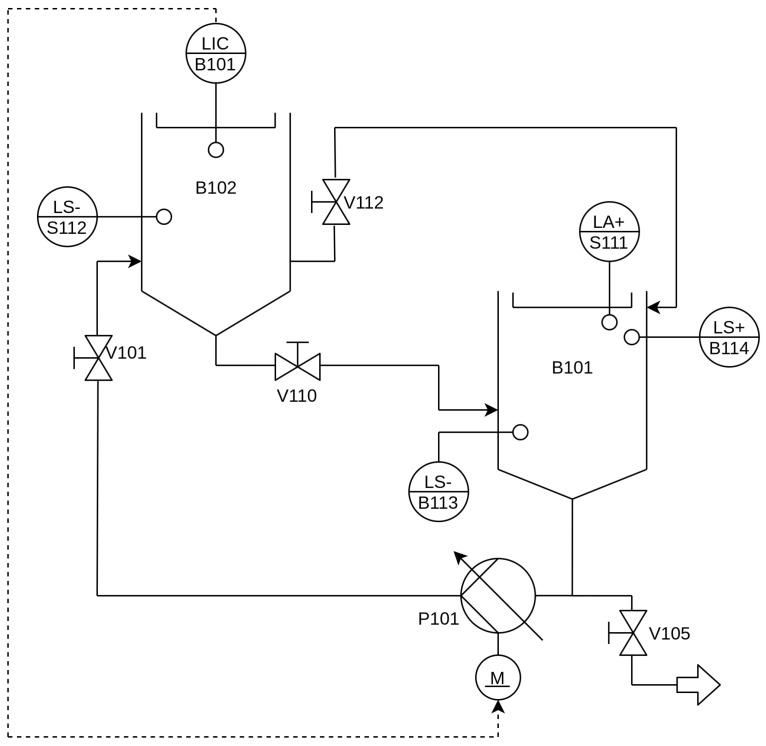
PI&D level diagram.

The objective of this research project is to address the growing demand for automation in industrial processes by implementing distributed control systems to enhance production and service efficiency. To achieve this, a constrained DMC strategy based on the IEC-61499 standard and the OPC-UA protocol was developed, aiming to achieve a high degree of distributed control. For the case study, the FESTO™ MPS-PA Compact Workstation, a versatile system capable of simulating various processes, was chosen as the experimental platform. Specifically, the liquid-level process within the workstation was selected as the focus of the study. The process involves a pump (P101) transferring fluid from one tank (B101) to another tank (B102) through a piping system. The level of fluid in the receiving tank (B102) is monitored using an ultrasonic analog sensor (B101) at the LIC measuring point. The PI&D diagram in Figure 6 depicts the components of the level process.

The goal is to maintain the fluid level at a specific setpoint, even in the presence of disturbances or changes in the setpoint. By implementing the constrained DMC strategy on the chosen platform, the research aims to demonstrate the effectiveness and applicability of distributed control in industrial automation, showcasing the potential for improved process control and operational efficiency.

The control strategy implemented in this research project is based on the IEC-61499 standard, which provides a framework for distributed control systems. By leveraging the IEC-61499 standard, the control logic can be modularized into FBs, allowing for a more flexible and scalable control architecture. These FBs encapsulate the necessary algorithms and control parameters for regulating the liquid level process. The use of IEC-61499 enables the distributed deployment of control functionalities across different devices within the FESTO™ MPS-PA Compact Workstation, facilitating efficient communication and coordination between the various components.

To enable real-time monitoring and data exchange between the control system and the process, the OPC-UA protocol is employed. OPC-UA serves as the communication framework that ensures interoperability and seamless integration between different devices and software applications. In this research, OPC-UA enables the monitoring of critical process variables such as level sensor, predicted output of the plant, control signal, and assigned setpoint. The utilization of OPC-UA enhances connectivity and data accessibility, enabling effective supervision and control of the level process.

By combining the power of the IEC-61499 standard for distributed control and the OPC-UA protocol for monitoring and data exchange, this research project aims to showcase the advantages of integrating these technologies into industrial automation. The IEC-61499-based control strategy provides a modular and scalable approach, while OPC-UA enables seamless communication and interoperability. Together, they form a robust foundation for achieving distributed control in the automation of industrial processes. The integration of these technologies within the FESTO™ MPS-PA Compact Workstation creates an experimental environment that allows for comprehensive evaluation and analysis of the proposed control strategy’s performance, effectiveness, and applicability in real-world industrial scenarios.

### 4.1. Software and Hardware Platform

In order to comply with the IEC-61499 standard for distributed control applications, the 4diac-IDE and 4diac-FORTE was utilized. The 4diac-IDE provides a development environment that supports the modeling of applications adhering to the IEC-61499 standard, which can then be downloaded and deployed on distributed field devices. This allows for the seamless transfer of control logic to the target devices based on the specifications defined by IEC-61499. For the runtime platform, the 4diac-FORTE was selected, which is a lightweight and portable implementation designed for small embedded control devices, typically with 16/32-bit architectures implemented in C++, and offers support for different types of FBs, Basic Function Blocks (BFBs), Service Function Blocks (SFBs), adapters, and sub-applications. To create the experimental setup, the Texas Instruments BBB was chosen as the embedded system that offers a low-cost development platform that is widely supported by a community of developers and hobbyists. It possesses analog input pins and PWM output capabilities, making it suitable for interfacing with the FESTO™ MPS-PA Compact Workstation and implementing the constrained DMC control strategy.

**Figure 7:**
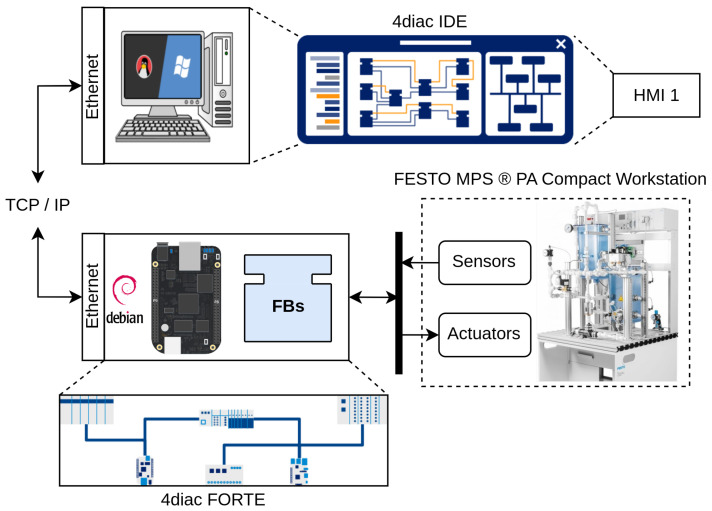
Software and hardware distribution.

Figure 7 shows the distribution of software and hardware used in the case study, which consists of an engineering station (PC) for modeling the FBs and monitoring the distributed control system, a BBB as an embedded controller, and finally the FESTO MPS-PA Compact Workstation, which serves as the physical plant being controlled. This configuration enables the distributed control system implementation using IEC-61499 standard and OPC-UA protocol with experimental station FESTO, providing a comprehensive platform for studying and evaluating the performance of the constrained DMCs in a practical industrial setting.

### 4.2. Function Blocks Methodology

The FB programming process based in IEC-61499 involved creating the FB structure in the 4diac-IDE environment. Initially, an empty FB structure was generated and subsequently exported as .cpp and .h files to 4diac-FORTE. These files were then edited to incorporate the necessary algorithms and user libraries for the specific functionality of each FB. Se crearon 3 FB: ANALOG_INPUT FB or reading the level sensor, DMC_CONTROL FB d where the algorithm of the constrained DMC is located, and OPC_UA_CLIENTE FB, which is an OPC-UA client.

In order to read the analog pins of the BBB card within the IEC-61499 architecture, a custom FBs was designed and implemented. This FB was specifically developed to provide access to the desired analog input pin of the BBB. In the same way, a dedicated FB was developed to implement the constrained DMC control strategy. This FB contain the necessary algorithms, parameters, and interfaces to execute the constrained MPC calculations and generate appropriate control signals. The constrained DMC FB receive the necessary input data, such as setpoints, and compute the optimal control actions based on the specified constraints and objectives. Finally, to enable communication and data exchange between the control system and external applications or monitoring tools, a Publisher Client FB, server, and subscriber clients were implemented. The client FB is able to send the constrained DMC variables to the server. The server provides the necessary functionality to expose process variables to external devices facilitating real-time monitoring and control.

#### 4.2.1. Analog Input Function Block

**Figure 8:**
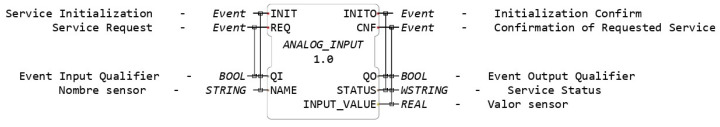
FB ANALOG_INPUT.

To utilize the analog input functionality of the BBB card under the 4diac methodology, the corresponding FB called ANALOG_INPUT was designed. This FB is responsible for reading the level sensor values and compares them with the predicted output values by the control algorithm implemented in the DMC_CONTROL FB. Within the FB structure, various input and output data ports were defined to facilitate communication. One such input port is the STRING-type data named NAME, which allows the user to specify the name of the sensor being read. Additionally, a REAL-type data output port named INPUT_VALUE was included, which provides the actual sensor value. The designed FB is shown in Figure 8.

#### 4.2.2. Constrained DMC Function Block

By means of the mathematical model represented as a transfer function of the level process and the minimization of the objective Function (2) with and without restrictions, it was possible to obtain the control signal, which was sent to the actuator or pump. These elements constitute the DMC algorithm implemented in the FB.

**Figure 9:**
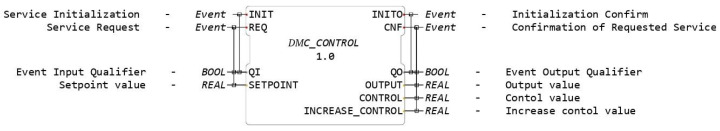
FB for constrained DMC.

The FB called DMC_CONTROL, designed for implementing the constrained DMC algorithm, as depicted in Figure 9, plays a crucial role in executing the control strategy and includes various REAL-type data inputs and outputs port required for effective control. One such input is the setpoint, which reads the desired setpoint value for the liquid level. The FB also features three outputs: OUTPUT-ym(k) which represents the predicted output of the level plant, CONTROL-u(k) which represent the actual control signal send to actuator, and INCREASE_CONTROL-Δu(k) which denotes the change in control signal at each iteration.

**Figure 10:**
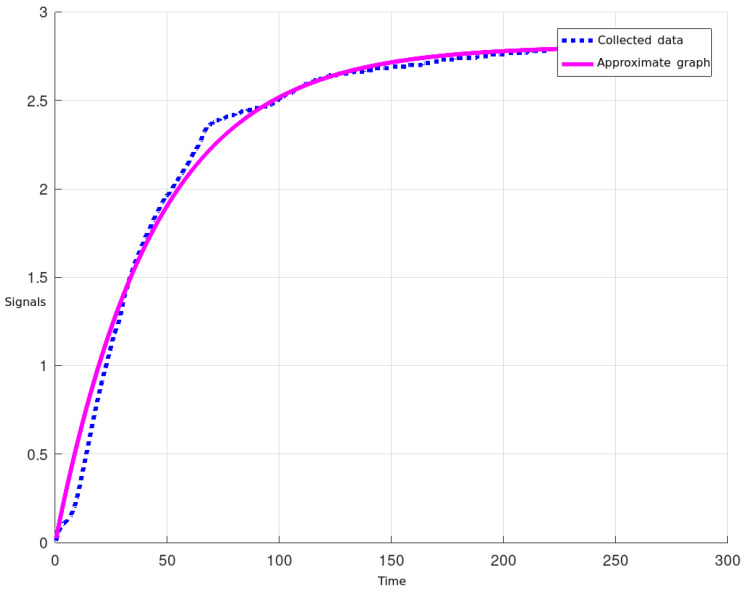
Response to a step input.

The implementation of the constrained DMC algorithm within the FB followed a systematic approach. First, the mathematical model of the level plant was determined using the exponential regression method. Experimental data capturing the level system’s response to a step input were collected and, based on these data, the best-fitting first-order transfer function was derived. This mathematical model serves as the basis for the constrained DMC algorithm implementation. The plot of the equation of the curve that best fits the collected data is shown in Figure 10.

The continuous and discrete transfer functions obtained are $G\left(s\right)=\frac{0.6718}{44.05s+1}$ and $\;G\left(z\right)=\frac{0.006073}{z-0.991}$, respectively, and a prediction horizon P=15, control horizon N=5, δ=0.2 and λ=10 were also established. Once the parameters were defined, the DMC algorithm was implemented with and without constraints. The calculation of the predicted output signal and the control signal implemented in the FB is shown below.

1. Predicted output signal ym(k).
  Considering the discrete time transfer function, the difference equation was obtained and is the predicted output signal by the controller.
  (9) ym(k)=0.006073u(k−1)+0.991y(k−1)
2. Control signal variation Δu(k).
  The matrix *G* is formed with the coefficients $g_{i}$ of the unit step response of the discrete-time transfer function.
  $$G=\left[\begin{array}{ccccc} 0 & 0 & 0 & 0 & 0\\ 0.0060727 & 0 & 0 & 0 & 0\\ 0.012091 & 0.0060727 & 0 & 0 & 0\\ 0.018054 & 0.012091 & 0.0060727 & 0 & 0\\ 0.023964 & 0.018054 & 0.012091 & 0.0060727 & 0\\ 0.02982 & 0.023964 & 0.018054 & 0.012091 & 0.0060727\\ 0.035623 & 0.02982 & 0.023964 & 0.018054 & 0.012091\\ 0.041374 & 0.035623 & 0.02982 & 0.023964 & 0.018054\\ 0.047072 & 0.041374 & 0.035623 & 0.02982 & 0.023964\\ 0.052719 & 0.047072 & 0.041374 & 0.035623 & 0.02982\\ 0.058316 & 0.052719 & 0.047072 & 0.041374 & 0.035623\\ 0.063861 & 0.058316 & 0.052719 & 0.047072 & 0.041374\\ 0.069357 & 0.063861 & 0.058316 & 0.052719 & 0.047072\\ 0.074802 & 0.069357 & 0.063861 & 0.058316 & 0.052719\\ 0.080199 & 0.074802 & 0.069357 & 0.063861 & 0.058316 \end{array}\right]$$
  For the case of the unconstrained DMC, the matrix K calculated according to Equation (4) is:
  $$K=\left[\begin{array}{ccccccccccccccc} 0.0000 & 0.0001 & 0.0002 & 0.0004 & 0.0005 & 0.0006 & 0.0007 & 0.0008 & 0.0009 & 0.0011 & 0.0012 & 0.0013 & 0.0014 & 0.0015 & 0.0016 \end{array}\right]$$
  and the value of Δu(k) is the first value of the vector Δu calculated by Equation (4).
  The QP Algorithm is used to compute Δu(k) for the constrained DMC where the objective function set is Equation (5); from this, we obtain matrix Equation (6) and matrix Equation (7). The restrictions implemented are:
  Increased control Δu(k) should not exceed the values of 0.1 y −0.1 when there is an increase or decrease in the setpoint, respectively.
  $$\left[\begin{array}{c} I\\ \cdots \\ -I \end{array}\right]\Delta u\leq \left[\begin{array}{c} 1*0.1\\ \cdots \\ -1*-0.1 \end{array}\right]$$
  Control signal u(k) must not exceed the values of 13.5 y 0.0 when there is an increase or decrease in the setpoint, respectively.
  $$\left[\begin{array}{c} T\\ \cdots \\ -T \end{array}\right]\Delta u\leq \left[\begin{array}{c} 1*13.5-1u(k-1)\\ \cdots \\ -1*0.0+1u(k-1) \end{array}\right]$$
  Plant predicted output signal ym(k) must not exceed the values of 5.5 y 0.0 when there is an increase or decrease in the setpoint, respectively.
  $$\left[\begin{array}{c} G\\ \cdots \\ -G \end{array}\right]\Delta u\leq \left[\begin{array}{c} 1*5.5-f\\ \cdots \\ -1*0.0+f \end{array}\right]$$
  For the QP Algorithm implementation the matrix (6) will be C and the matrix (7) will be $c^{T}$;
3. Control output u(k).
  The control signal u(k) calculated is:
  u(k)=u(k−1)+Δu(k)

#### 4.2.3. OPC-UA Client Function Block

The publishing client implemented as an FB called OPC_UA_CLIENT is responsible for transmitting essential signals including the setpoint, control signal u(k), output signal ym(k), and sensor reading to the server. This publishing client plays a crucial role in providing real-time data updates to other components of the distributed control system. The publishing client FB can easily access and write the essential control signals in the server. In addition, it can utilize the necessary OPC-UA protocol functionalities like exploring the server OPC-UA address space, select nodes for writing, establish publications, and write data to the server in the desired nodes.

**Figure 11:**
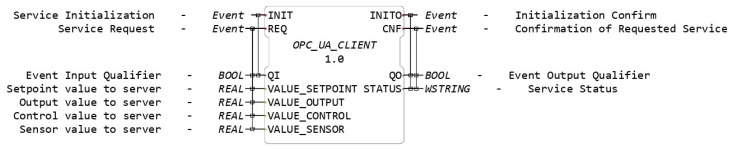
FB OPC-UA client.

The FB represented in Figure 11 was designed to implement a client that will publish the different values of the constrained DMC variables on the server. It contains four REAL type data inputs named VALUE_ SETPOINT, VALUE_OUTPUT, VALUE_CONTROL, and VALUE_SENSOR, which read the values of the setpoint, ym(k), u(k), and sensor, respectively.

### 4.3. OPC-UA Methodology

**Figure 12:**
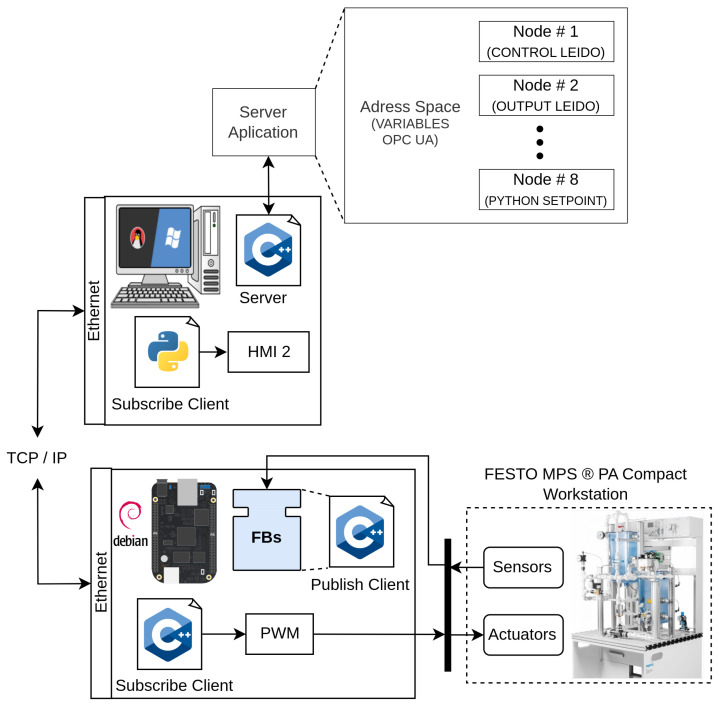
OPC-UA application scenario.

Three clients and one server were created for OPC-UA communication in the distributed control system implementation, each client serving a specific purpose. A first publishing client was implemented in the OPC_UA_FB, another subscriber client was implemented in the engineering station PC (HMI-2) and, finally, a third subscriber client was implemented in the BBB without IEC-61499 standard, which sends the control signals u(k) in the form of Pulse Width Modulation (PWM) to the actuator. The OPC-UA application also has a server in which information about the DMC variables is stored, distributed, and supplied. The application scenario OPC-UA is depicted in Figure 12. This scenario involves the exchange of various signals crucial for the constrained DMC implementation, including setpoint, Control signal u(k), Output signal ym(k), and Sensor readings.

To realize the OPC-UA application, two libraries were utilized: the open62541 library for C++ and the FreeOpcUa library for Python. These libraries served as the foundation for developing OPC-UA functionality, facilitating the establishment of communication channels, and enabling the seamless exchange of data between different devices and systems, providing robust support for implementing OPC-UA servers and clients within the distributed control systems. By leveraging the open62541 library in C++ and the FreeOpcUa library in Python, the OPC-UA application within the constrained DMC implementation facilitated seamless communication and data exchange. This integration allowed for real-time monitoring and control of the DMC process, enabling efficient coordination between the control algorithm, data acquisition, and external systems.

#### 4.3.1. OPC-UA Server

The server OPC-UA, which is implemented on the PC for monitoring the process variables, plays a crucial role in the overall architecture of the distributed control system. It serves as the central component responsible for receiving and managing various signals from the publishing client OPC-UA FB. These signals include important parameters such as the Setpoint, Control signal u(k), Output signal ym(k), and Sensor readings. By collecting and processing this information, the server enables efficient communication and coordination among different parts of the distributed control systems.

The server OPC-UA incorporates specific routines to ensure its proper functioning. These routines include controlling the life cycle of the server, which involves initializing it, executing the necessary operations, and ultimately deleting it when no longer needed. Additionally, the server configuration involves setting up the communication network port by specifying the hostname IP address and network port to establish seamless communication with other devices and clients within the system.

**Figure 13:**
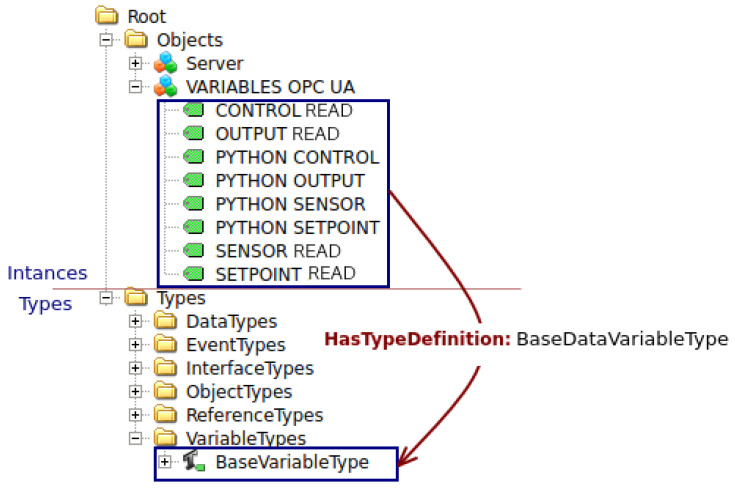
Example of address space modeling.

One of the key aspects of the server implementation is the creation of a new node within the OPC-UA Address Space. This node serves as a container for various objects, known as “OPC-UA VARIABLES”, which encapsulate specific data and functionality required for the control process. For the constrained DMC implementation, the server adds crucial variables such as “SETPOINT READ”, “CONTROL READ”, “OUTPUT READ”, “SENSOR READ”, “PHYTON SETPOINT”, “PHYTON CONTROL”, “PHYTON OUTPUT”, and “PHYTON SENSOR”, as shown in Figure 13. These variables act as the bridge between the control algorithm and the physical process, allowing seamless exchange and updating of relevant data.

By reading the values associated with the specific variables denoted by NodeClass, the server gains access to information. For example, “SETPOINT LEÍDO” provides the desired setpoint value, “CONTROL LEÍDO” represents the current value of the control signal u(k), “OUTPUT LEÍDO” captures the current value of the output signal ym(k), and “SENSOR LEÍDO” monitors the level sensor readings. On the other hand, the server also exposes certain variables, such as “PYTHON SETPOINT”, “PYTHON CONTROL”, “PYTHON OUTPUT”, and “PYTHON SENSOR”, to other clients within the system.

These variables allow other clients to read the values stored in the server, enabling functionalities such as real-time visualization and monitoring of the control process through a Human Machine Interface (HMI) or any other client application. This distributed control systems approach, facilitated by the server’s implementation, provides flexibility, scalability, and efficient control capabilities that are not easily achievable with traditional control devices like PLCs. The proper functioning of the server was verified using a UAExpert client. UAExpert is a powerful Graphical User Interface (GUI) tool widely used in OPC-UA applications for testing and monitoring purposes. It provides a comprehensive view of the OPC-UA server and allows users to interact with the server, read and write variables, and monitor the communication status.

#### 4.3.2. OPC-UA Clients

Two subscribing clients were implemented: The first subscribing client, implemented with the open62541 library in the BBB, reads the value of the control signals u(k) published in the server OPC-UA. This value is then utilized to generate the appropriate PWM signal, which controls the actuator operation. The second subscribing client, implemented on the PC, serves the purpose of visualizing all constrained DMC signals in a HMI for monitoring and control purposes.

To achieve the desired functionality, several routines were implemented for the creation of these clients. The first routine involves exploring the OPC-UA address space, which allows the clients to navigate through the available nodes and identify the desired variables for reading. The next step involves selecting the appropriate nodes for these operations, specifying which variables will be accessed and read data from the desired nodes. Once the nodes are selected, the clients call subscription services and establish access to the server’s data from the desired nodes. This enables the clients to receive real-time updates and maintain synchronized communication with the server. Finally, with access to the server’s data, the subscribing clients can visualize and control specific processes in real-time, allowing for effective monitoring and adjustment of the control system’s behavior. These routines ensure seamless communication and enable the clients to interact with the server and exchange data efficiently.

**Figure 14:**
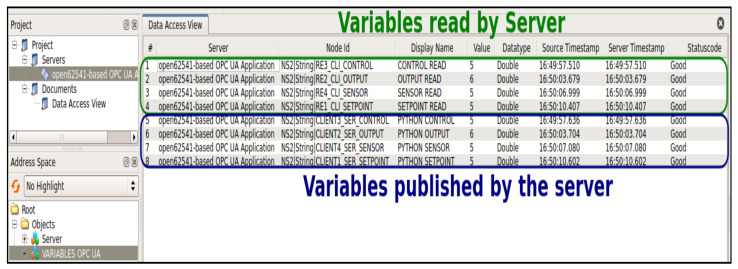
UAExpert OPC-UA client.

In Figure 14, the UAExpert client interface is depicted, showcasing a successful communication between the server, OPC-UA client FB, and UAExpert client. By utilizing UAExpert, operators and developers can easily monitor and validate the server’s behavior, ensuring smooth communication and data exchange between the different components of the distributed control systems.

## 5. Results

**Figure 15:**
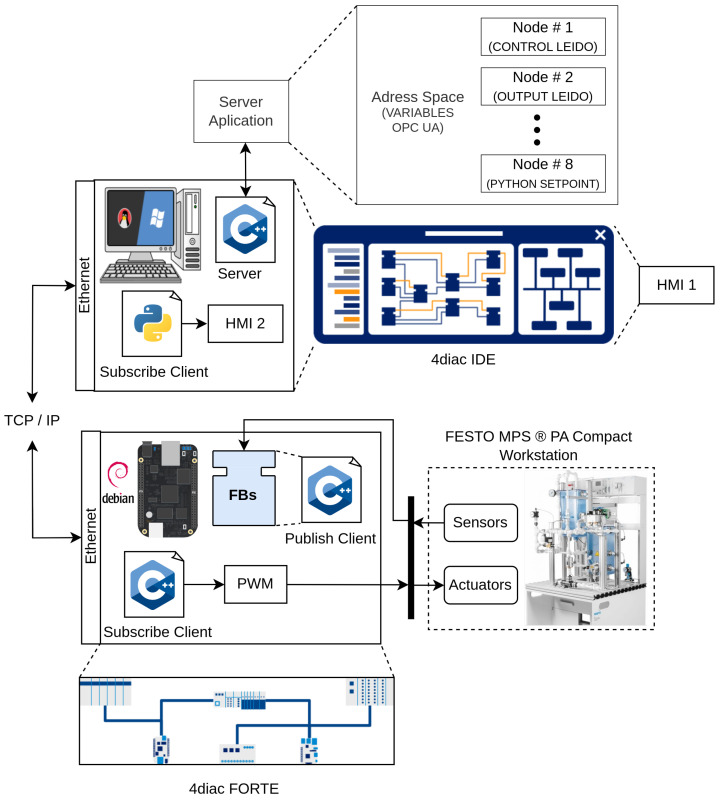
IEC-61499 and OPC-UA methodology distribution.

The implementation of the constrained DMC based on the IEC-61499 standard and the OPC-UA protocol involved a distribution of software and hardware components, as depicted in Figure 15. The PC played a crucial role in the execution of several components:

- 4diac-IDE: This powerful IDE was utilized for the modeling and interconnection of the FBs with SIFBs. It provided a user-friendly interface for designing and configuring the control logic of the system. Additionally, it ran an HMI-1 application responsible for visualizing the behavior of process variables and enabling the adjustment of the setpoint;
- OPC-UA server: Acting as a central hub, the OPC-UA server played a vital role in the implementation. It was responsible for storing the variable data, ensuring secure and reliable communication between the various components. The server allowed the different clients to access the stored data over the network, facilitating real-time control and monitoring;
- OPC-UA client: Another component running on the PC was an HMI-2 application, providing real-time visualization of the process variables. This client application allowed operators and users to monitor the system’s behavior, view relevant data, and make informed decisions based on the displayed information.

On the BBB card, direct interaction with the sensor and actuator of the level plant occurred, along with the execution of specific components:

- 4diac-FORTE: This module encompassed the source code of all implemented Function Blocks (FBs), enabling the execution of control logic on the BBB card. It served as a runtime platform, ensuring the correct functioning of the FBs and their integration with the hardware components of the level plant. The 4diac-FORTE implementation allowed for efficient and reliable execution of the DMC control algorithm on the low-cost BBB platform;
- OPC-UA client: The BBB card also hosted an OPC-UA client responsible for sending the Pulse Width Modulation (PWM) signal to the pump, thus controlling its operation. This client component facilitated the seamless communication between the BBB card and the OPC-UA server, allowing for the transmission of control signals and ensuring synchronized control of the physical process.

By employing this distributed setup, the implementation achieved effective collaboration between the PC and the BBB card, facilitating real-time communication, control, and visualization of the constrained DMC system. The successful integration of IEC-61499 FB, coupled with the utilization of OPC-UA protocols, enabled efficient and distributed control of the level plant. This approach demonstrated the feasibility of implementing advanced control algorithms using low-cost devices, opening up possibilities for cost-effective automation solutions in various industrial domains.

**Figure 16:**
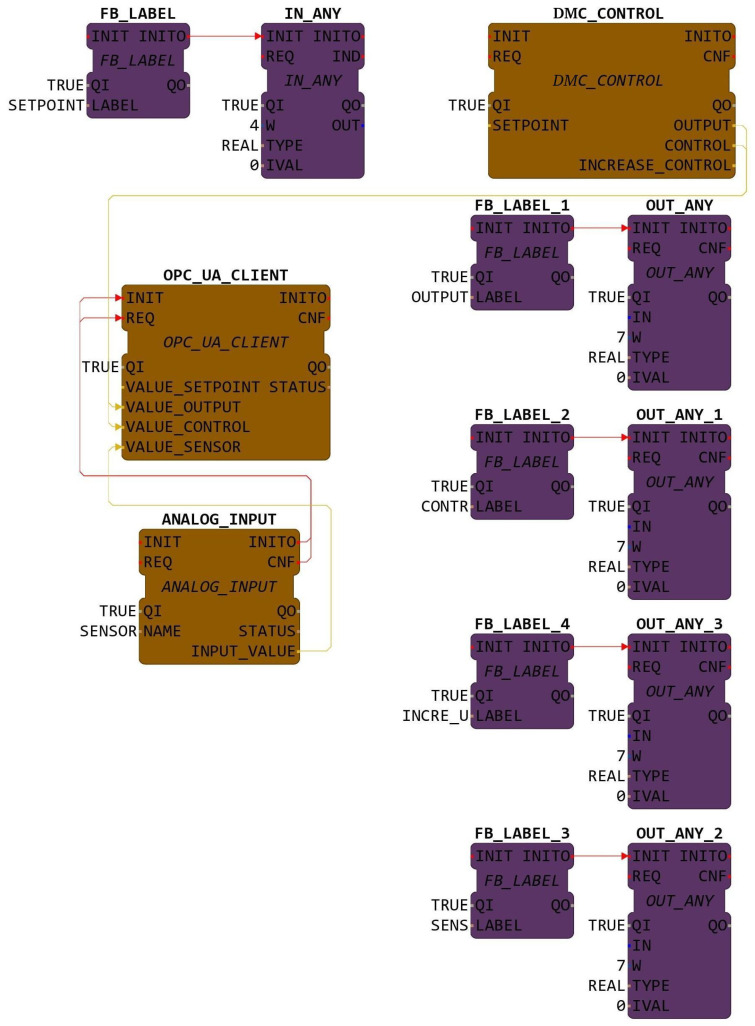
Modeling of FBs in 4diac-IDE.

The modeling of the FBs of the constrained DMC implemented in the 4diac-IDE is represented in Figure 16. The first device will be the HMI-1 station, represented in purple color. This device will allow the manipulation of the desired setpoint; additionally, it will indicate the values of plant output ym(k), control signal u(k), sensor value, and increment in the control signal Δu(k). The second device will be the BBB card, represented in brown color, which contains the FBs of the level sensor, constrained DMC algorithm, and publishing client.

### 5.1. Validation Test

To validate the effectiveness of the constrained DMC algorithm, two tests were conducted, each with a different setpoint value. The objective of the performance tests was to verify the proper functioning of the DMC algorithm. The first parameter verified was that the output signal ym(k) reaches the established setpoint values. The second parameter verified was that the signals of the DMC algorithm do not exceed the values established as maximum and minimum limits.

One such analyzed parameter was the maximum and minimum increase in control signal $\Delta u_{\max}$. This parameter plays a crucial role in maintaining stability and preventing abrupt changes in the control signal u(k). By setting an appropriate $\Delta u_{\max}$ value, the constrained DMC algorithm can effectively manage the control signal u(k) while ensuring smooth operation. Another critical parameter monitored during the tests was the control signal u(k). The behavior and characteristics of the control signal were analyzed to assess its effectiveness in regulating the system and achieving the desired setpoint. Similarly, the parameter of the predicted output signal ym(k) was analyzed and restricted to a certain value, thus ensuring the proper functioning of the distributed control systems.

Through the analysis of the output signal, the maximum increase in control, and the control signal, the performance and robustness of the DMC algorithm could be evaluated. These tests provided valuable insights into the behavior of the system under different setpoint conditions, facilitating the optimization and refinement of the control strategy if required.

#### 5.1.1. Functional Test 1

In functional test 1, a setpoint value of 4.5 L was assigned to evaluate the performance of the constrained DMC algorithm within the context of the IIoT paradigm using the IEC-61499 standard. The objective was to assess the algorithm’s ability to regulate the system and achieve the desired setpoint while adhering to specific constraints.

During the test, the behavior of the system was closely monitored and analyzed. It was observed that the predicted output signal, denoted as ym(k), successfully reached the desired value of 4.5 L. This result indicated that the constrained DMC algorithm effectively regulated the system to achieve the specified setpoint.

**Figure 17:**
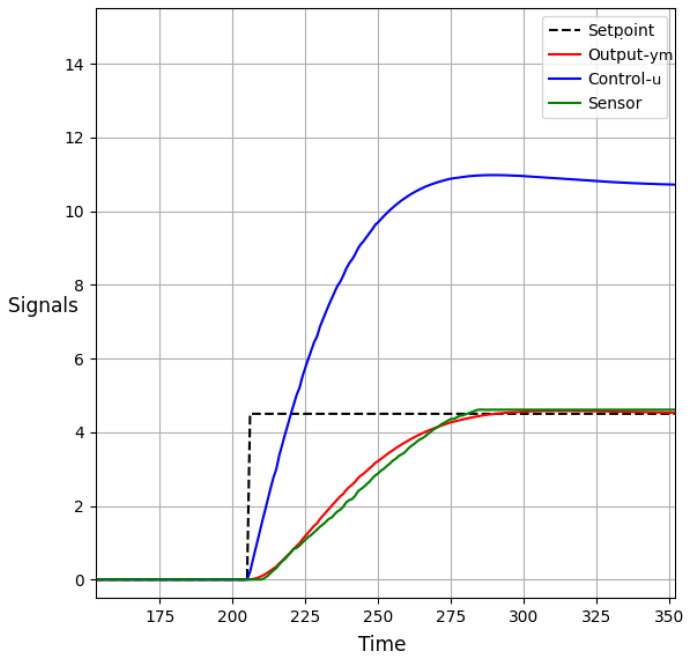
Constrained DMC signals. Setpoint value 4.5 L.

To further evaluate the performance, the test results and system behavior are depicted in Figure 17 (HMI-2). Illustrates the relationship between time and the predicted output signal (ym(k)). As shown, the predicted output signal steadily approaches and eventually reaches the desired setpoint value of 4.5 L. Importantly, it is also observed that the predicted output signal does not exceed the upper limit of 5.5 L, demonstrating the algorithm’s ability to maintain the system within the desired range.

Furthermore, Figure 17 also displays the control signal (u(k)) generated by the algorithm during the test. It can be seen that the control signal remains within the acceptable range and does not exceed the maximum value of 13.5 units. This indicates that the constrained DMC algorithm effectively manages the control signal to prevent unnecessary stress on the actuators while regulating the system.

**Figure 18:**
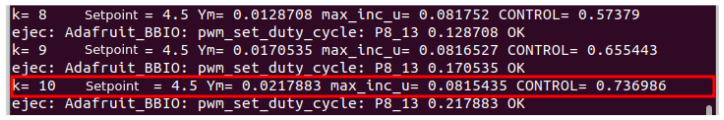
Constrained DMC at time *k*. In this image, we can see the set-point and the predicted output signal, denoted as $y_{m}\left(k\right)$, as well as the increase in the control signal ($\Delta u_{\max}$) and the control signal for Test 1.

Additionally, it is crucial to ensure that the control signal increment, denoted as $\Delta u_{\max}$, complies with the specified restriction of 0.1. Figure 18 (HMI-1) provides a graphical representation of the control signal increment over time. It clearly shows that the increase in the control signal ($\Delta u_{\max}$) remains well below the established restriction of 0.1, indicating that the algorithm effectively manages and limits the control signal increment to prevent abrupt changes in system behavior.

The successful performance of the constrained DMC algorithm in functional test 1 highlights its capability to optimize system operation while adhering to specific constraints. By leveraging the IEC-61499 standard, OPC-UA protocol, and low-cost devices, advanced control strategies can be seamlessly integrated into industrial processes within the IIoT paradigm. This integration leads to improved system performance, enhanced resource utilization, increased overall productivity, and consideration of system limitations and constraints.

#### 5.1.2. Functional Test 2

In functional test 2, a setpoint value of 6.0 L was assigned to evaluate the performance of the constrained DMC algorithm. The objective of this test was to assess the system’s behavior when the desired setpoint was set to a value that exceeded the constraint of 5.5 L. In this test, it was observed that the predicted output signal ym(k) did not reach the desired value of 6.0 L. This behavior was due to the constraint imposed on the DMC algorithm, which limited the predicted output signal to a maximum of 5.5 L. Despite the deviation from the desired value, the control algorithm ensured that the predicted output signal ym(k) remained within the specified constraint.

Similar to functional test 1, the control signal u(k) was kept within a safe range to maintain stable operation. The control signal did not exceed 13.5 units, indicating that the control algorithm effectively regulated the system without causing excessive control actions. Furthermore, the restrictions on the control increment Δu(k) helped maintain smooth transitions in the predicted output signal ym(k), preventing sharp increases or decreases. As in functional test 1, maximum control increment $\Delta u_{\max}$ was limited to 0.1 units for incrementing the setpoint and −0.1 units for decrementing the setpoint. By imposing these restrictions, the predicted output signal’s behavior remained controlled and avoided sudden variations.

**Figure 19:**
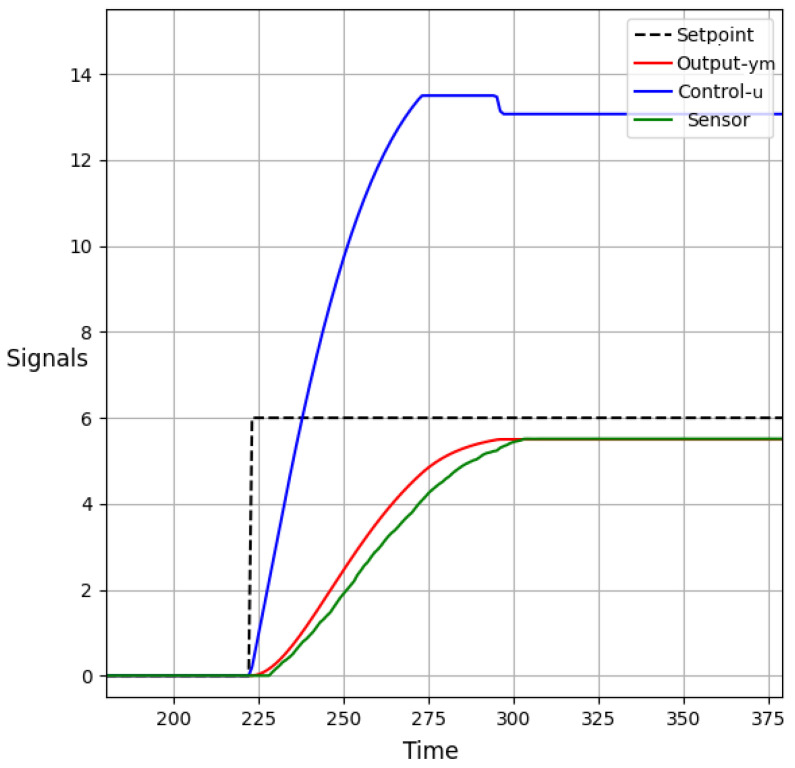
Constrained DMC signals.

**Figure 20:**
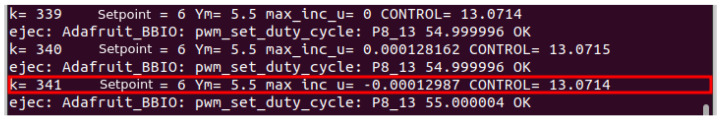
Constrained DMC at time *k*. In this image, we can see the set-point and the predicted output signal, denoted as $y_{m}\left(k\right)$, as well as the increase in the control signal ($\Delta u_{\max}$) and the control signal for Test 2.

The results and behavior of the system during functional test 2 are illustrated in Figure 19 (HMI-2) and Figure 20 (HMI-1). These visual representations provide valuable insights into the system’s performance, enabling further analysis and evaluation of its response under different operating conditions.

#### 5.1.3. Benefits of the Constrained DMC Controller

**Figure 21:**
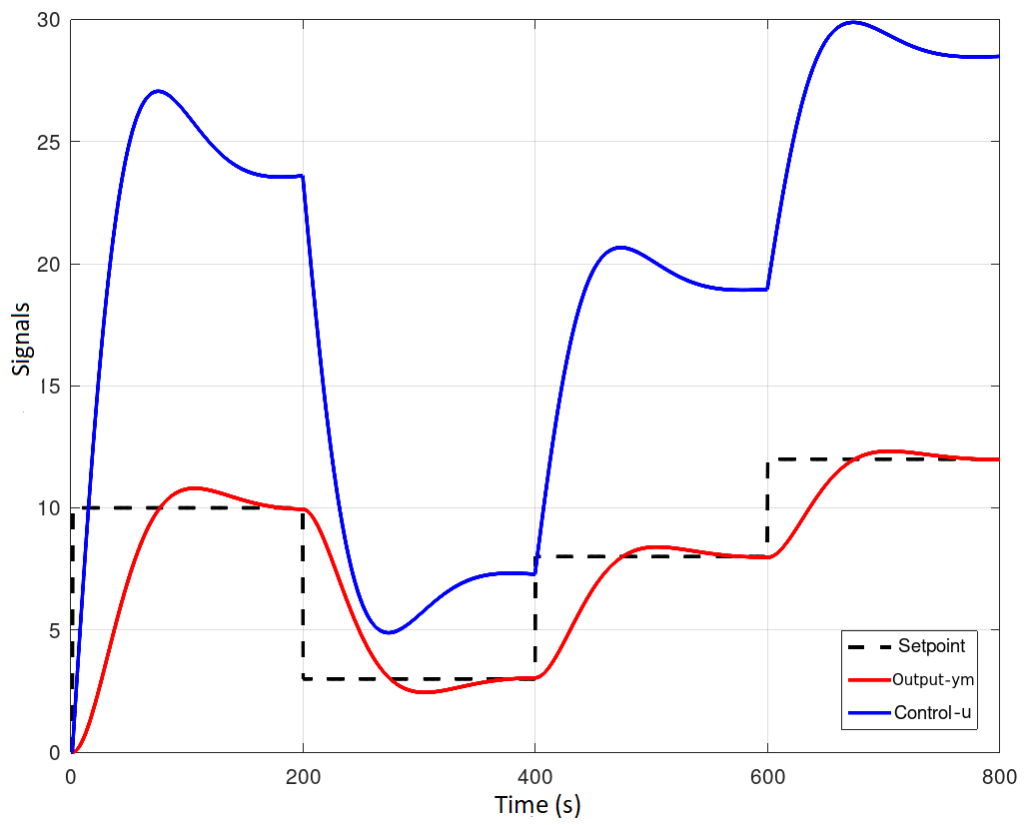
Unconstrained DMC signals.

**Figure 22:**
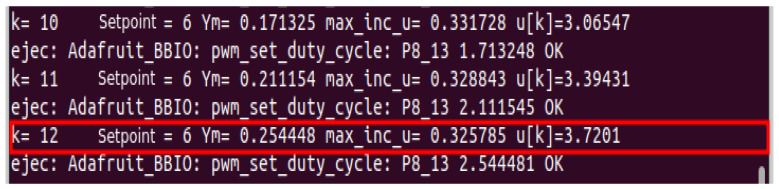
Unconstrained DMC at time *k*. In this image, we can see the set-point and the predicted output signal, denoted as $y_{m}\left(k\right)$, as well as the increase in the control signal ($\Delta u_{\max}$) and the control signal.

To verify the benefits of the constrained DMC controller, first, an unconstrained DMC controller was implemented as shown in Figure 21 and Figure 22. Several setpoints equal to and greater than 10 L were assigned, the control increment Δu(k) reaches a value of 0.325785 at a selected instant *k*, which places excessive demands on the pump reducing its life cycle; in addition, it is observed that the predicted output signal ym(k) reaches values above 10 L which are out of range of the tank.

**Figure 23:**
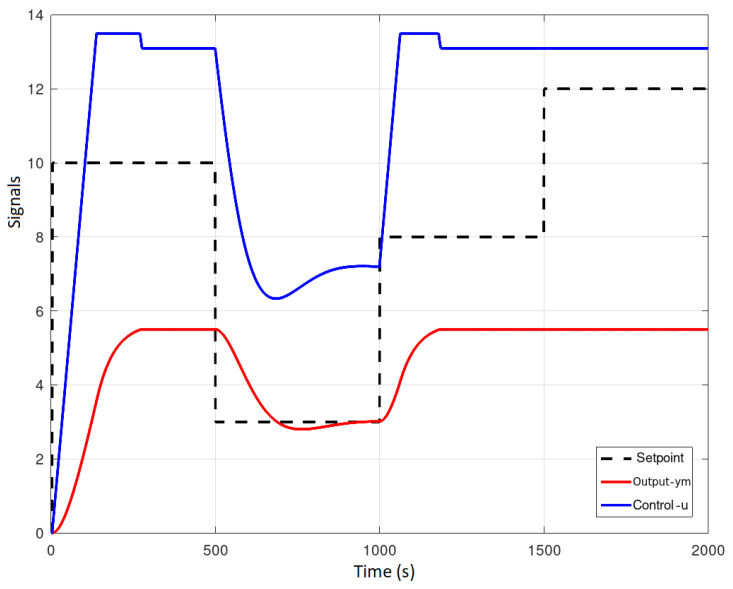
Constrained DMC signals.

**Figure 24:**
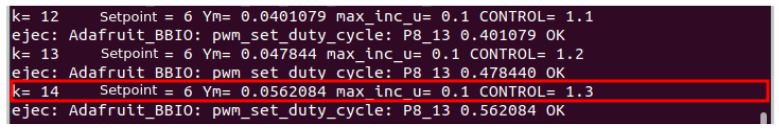
Constrained DMC at time *k*. In this image, we can see the set-point and the predicted output signal, denoted as $y_{m}\left(k\right)$, as well as the increase in the control signal ($\Delta u_{\max}$) and the control signal.

To avoid the aforementioned drawbacks, the constrained DMC was implemented as shown in Figure 23 and Figure 24. The control increment Δu(k) no longer reaches the value of 0.3257 at instant *k*, it is now restricted to a value of 0.1000, thus avoiding premature pump wear; furthermore, it is observed that the output signal ym(k) does not exceed the value of 10 L since the predicted output ym(k) and control u(k) signals are restricted to values of 5.5 L and 13.5 units, respectively.

## 6. Discussion

Correct operation of the distributed control system implemented in the level plant of the FESTO™ MPS-PA Compact Workstation was achieved. The objective of the performance tests was to assess the effectiveness of the constrained DMC algorithm in reaching the assigned setpoints and adhering to the specified constraints.

In the Dynamic Matrix Control (DMC) method, approximating disturbances as constant over the prediction horizon is a common assumption made for several reasons. This assumption simplifies the control algorithm, making it more computationally efficient and easier to implement. Additionally, it allows the controller to effectively handle disturbances that change relatively slowly compared to the control system’s dynamics.

The rationale behind assuming constant disturbances lies in practical considerations and the desire to strike a balance between accuracy and simplicity. In many industrial processes, disturbances may not exhibit rapid and drastic changes over short time intervals. Instead, disturbances often manifest as slow variations or steady-state offsets, which can be effectively approximated as constant over a certain prediction horizon.

The primary objective of the DMC method is to counteract the effect of disturbances and maintain the process variable at the desired setpoint. In many practical applications, disturbances often exhibit relatively slow variations or steady-state offsets. By assuming constancy over the prediction horizon, the controller can effectively compensate for these disturbances and steer the system towards the desired setpoint.

The constant disturbance assumption provides a reasonable compromise between accuracy and practicality; while it may not capture the exact dynamics of rapidly changing disturbances, it remains a robust and effective approach for scenarios where disturbances evolve gradually and have a slower rate of change.

Under what conditions is this assumption valid? The constant disturbance approximation is applicable in scenarios where the disturbances have a relatively slow rate of change and do not significantly impact the control system dynamics within the prediction horizon. For instance, in processes where disturbances arise from slow environmental changes, gradual variations in raw material properties, or slowly evolving external conditions, the constant disturbance assumption can provide a reasonable approximation.

However, it is essential to acknowledge the limitations of this assumption. In situations where disturbances are highly dynamic or exhibit rapid fluctuations, the constant disturbance approximation may lead to inaccuracies in the control response. Furthermore, if the control system’s dynamics are sensitive to changes in disturbances, relying on this assumption could lead to suboptimal control performance.

In real-world applications, it is crucial to carefully evaluate the characteristics of the disturbances and the control system’s response dynamics to determine the appropriateness of the constant disturbance assumption. Robustness analysis and sensitivity studies can help assess the impact of this assumption on control performance under various disturbance scenarios.

The goal of maintaining the fluid level at a specific setpoint, even in the presence of disturbances or changes in the setpoint, can be achieved effectively with the DMC method. By incorporating the constant disturbance approximation, the controller can generate control actions that anticipate and counteract disturbances, ensuring the desired setpoint is maintained.

The results of the performance tests demonstrated that the DMC algorithm successfully met the setpoint objectives in each test. The signals used in the DMC algorithm, such as the predicted output signal (ym(k)) and the control signal (u(k)), remained within the desired ranges and did not exceed the predefined limits. This indicates that the constrained DMC algorithm effectively regulated the system while considering the constraints imposed by the system’s dynamics and operational limitations.

Comparing the results of the constrained DMC with an unconstrained DMC highlighted the benefits of incorporating constraints into the control algorithm. The use of constraints provided advantages for both the actuators and the plant supervisory staff. By considering control increment (Δu(k)), output (ym(k)), and control (u(k)) constraints, unnecessary stress on the actuators was prevented, minimizing the risk of damage to the plant. This enhanced the safety and reliability of the control system.

Furthermore, the successful implementation of the OPC-UA communication scenario between clients and servers enabled efficient manipulation and visualization of the DMC variables from any PC or embedded device connected to the local network of the distributed control system. This demonstrated the seamless integration and interoperability achieved through the utilization of OPC-UA. The ability to monitor and control the system in real time from various devices enhances the flexibility and accessibility of the control system.

The creation, modification, and modeling of Function Blocks (FBs) together with the Service Function Blocks (SFBs) based on the IEC-61499 standard proved to be successful. These FBs and SFBs formed the core components of the distributed control system, contributing to its robustness, reliability, and open-source nature. The adoption of the IEC-61499 standard facilitated the development of a modular and vendor-independent design, allowing for the flexible deployment and integration of control strategies in distributed industrial systems.

In conclusion, the implementation of the constrained DMC algorithm using the IEC-61499 standard and OPC-UA communication in the FESTO™ MPS-PA Compact Workstation plant showcased the feasibility and effectiveness of the proposed approach. The distributed control system demonstrated correct operation, achieving the assigned setpoints while adhering to the specified constraints. The benefits of incorporating constraints were evident in terms of actuator protection and the overall performance of the plant. Additionally, the successful implementation of the OPC-UA communication scenario showcased the seamless integration and real-time monitoring capabilities of the control system. The utilization of the IEC-61499 standard and the development of FBs and SFBs proved to be reliable and open-source solutions for implementing distributed control system paradigms.

The results of this research contribute to the advancement of industrial automation, particularly in the context of Industry 4.0 and IIoT applications. By leveraging the capabilities of the IEC-61499 standard, the use of advanced control techniques like DMC, and the interoperability provided by OPC-UA, manufacturing companies can achieve improved control performance, increased system flexibility, and efficient monitoring and control of industrial processes.

### Limitations of the Study

While the implemented approach utilizing the IEC-61499 standard, constrained DMC algorithm, and OPC-UA communication offers significant advantages for industrial control systems, there are certain limitations that should be considered.

Firstly, the study focused on the implementation and evaluation of the proposed approach in a specific plant, namely the FESTO™ MPS-PA Compact Workstation. The findings and conclusions may not be directly applicable to other industrial plants or systems with different characteristics, dynamics, or operational requirements. Further research and experimentation are necessary to validate the approach’s effectiveness in diverse industrial scenarios.

Secondly, the limitations of the low-cost devices used for data acquisition and actuator control should be acknowledged. While these devices provide cost-effective solutions, they may have limitations in terms of processing power, memory, and communication bandwidth. These limitations can potentially impact the scalability and performance of the control system, particularly when dealing with complex or high-speed processes. Therefore, careful consideration should be given to the selection and configuration of low-cost devices based on the specific requirements of the industrial application.

Thirdly, the constraints considered in the constrained DMC algorithm were limited to control increment (Δu(k)), output (ym(k)), and control (u(k)) constraints. Other types of constraints, such as state constraints or interaction constraints between different control loops, were not explicitly addressed in this study. Future research could explore the incorporation of additional types of constraints to further enhance the robustness and performance of the control system.

Lastly, while the utilization of the IEC-61499 standard and OPC-UA protocol facilitates interoperability and communication between devices and systems from different vendors, challenges may still arise in real-world implementations. Compatibility issues, differences in implementation interpretations, or specific configurations required by certain devices or software applications can pose challenges to achieving seamless integration. It is important to thoroughly test and validate the compatibility and interoperability of the components involved in the distributed control system to ensure successful deployment.

Understanding and addressing these limitations will contribute to the refinement and advancement of the proposed approach. Future research and practical implementations should consider these limitations while extending the application of the approach to various industrial settings and exploring additional strategies for effective control and optimization.

## 7. Conclusions

In this paper, we have successfully implemented a constrained DMC control algorithm using IEC-61499 FBs programmed on a low-cost BBB card. Through operation tests, we verified that the algorithm achieved the desired setpoint values while adhering to specified restrictions, ensuring motor longevity and enhancing safety measures for personnel and equipment in the FESTO™ MPS-PA Compact Workstation plant. The combination of DMC control, IEC-61499 FBs, and the OPC-UA protocol provided a robust and distributed control solution for industrial processes.

Comparing our research with previous studies, we have demonstrated the practicality and effectiveness of implementing the DMC algorithm using IEC-61499 FBs on a low-cost BBB card. By leveraging the capabilities of the BBB card and the flexibility of IEC-61499 FBs, we achieved precise control over dynamic systems while considering operational restrictions. This approach offers a cost-effective and scalable solution for industrial control systems, presenting an alternative to traditional control devices like PLCs.

The practical implications of our research are significant. The utilization of low-cost BBB cards and open-source tools like 4diac-IDE and 4diac-FORTE enables easier access to advanced control capabilities for small to medium-sized industries. The implementation of the constrained DMC algorithm showcases the potential of the IEC-61499 standard and the OPC-UA protocol in achieving distributed and efficient control over industrial processes. This can lead to improved productivity, energy efficiency, and reduced operational costs in various industrial applications.

While our research has provided valuable insights and promising results, it is important to acknowledge its limitations. The implementation was conducted on a specific level process using a limited number of variables and constraints. Further studies should explore the scalability and generalizability of this approach across different industrial systems and consider real-time experimentation on larger-scale setups. Additionally, future research should focus on integrating advanced technologies such as machine learning and artificial intelligence to enhance control performance and optimization in industrial processes.

In summary, our research has demonstrated the successful implementation of the constrained DMC control algorithm using IEC-61499 FBs on a low-cost BBB card. This approach offers practical and cost-effective control capabilities for industrial processes, contributing to the advancement of distributed control systems. By addressing limitations and exploring further implications, future research can continue to unlock the full potential of the IEC-61499 standard and the OPC-UA protocol in industrial applications.

## Data Availability

Not applicable.

## Abbreviations

The following abbreviations are used in this manuscript:

BBB	BeagleBone Black
BFB	Basic Function Block
COM	Component Object Model
CPPS	Cyber-Physical Production System
DCOM	Distributed Component Object Model
DCS	Distributed Control System
DMC	Dynamic Matrix Control
ECC	Event Execution Control
ERP	Enterprise Resource Planning
FB	Function Block
GQP	General Quadratic Programming
GUI	Graphical User Interface
HMI	Human Machine Interface
IDE	Integrated Development Environment
IEC	International Electrotechnical Commission
IIoT	Industrial Internet of Things
IoT	Internet of Things
IT	Information technology
MES	Manufacturing Execution System
MPC	Model Predictive Control
OPC	Open Platform Communications
OPC-DA	OPC Data Access
OPC-UA	OPC Unified Architecture
P&ID	Piping and Instrumentation Diagram
pHRI	Physical Human-Robot Interaction
PLC	Programmable Logic Controllers
PWM	Pulse Width Modulation
QP	Quadratic Programming
RPI	Raspberry Pi
SCADA	Supervisory Control and Data Acquisition
SFB	Service Function Block
SMPC	MPC Stochastic
